# Negative Nasopharyngeal SARS-CoV-2 PCR Conversion in Response to Different Therapeutic Interventions

**DOI:** 10.7759/cureus.21442

**Published:** 2022-01-20

**Authors:** Hassan Alwafi, Mohammed H Shabrawishi, Abdallah Y Naser, Ahmad M Aldobyany, Sultan A Qanash, Abdelfattah A Touman

**Affiliations:** 1 Pharmacology and Therapeutics, Umm Al-Qura University, Mecca, SAU; 2 Pulmonology Department, Al Noor Specialist Hospital, Mecca, SAU; 3 Faculty of Pharmacy, Isra University, Amman, JOR; 4 Pulmonology Department, King Abdullah Medical City, Mecca, SAU; 5 Department of Internal Medicine, King Saud bin Abdulaziz University for Health Sciences, Jeddah, SAU; 6 King Abdullah International Medical Research Center, King Abdulaziz Medical City, Jeddah, SAU

**Keywords:** novel coronavirus, infectious disease, sars-cov-2, hydroxychloroquine, covid-19

## Abstract

Background

The current management practices for patients with COVID-19 consist of infection prevention and supportive care. We aimed to explore the association between negative nasopharyngeal SARS-CoV-2 polymerase chain reaction (PCR) clearance and different therapeutic interventions.

Methods

This study is a retrospective cohort study of 93 patients who were admitted to a tertiary hospital in Saudi Arabia with a PCR confirmed diagnosis of COVID-19. There were three intervention subgroups (group A) (n = 45), which included those who received chloroquine or hydroxychloroquine (HCQ) only (A1), those who received chloroquine or HCQ in combination with azithromycin (A2), and those who received chloroquine or HCQ in combination with antiviral drugs with or without azithromycin (A3), as well as one supportive care group (group B) (n = 48). The primary and secondary endpoints were achieving negative SARS-CoV-2 nasopharyngeal PCR samples within five and 12 days from the start of the intervention, respectively.

Results

A median time of three days (interquartile range (IQR): 2.00-6.50) is needed from the time of starting the intervention/supportive care to the first negative PCR sample. There was no statistically significant difference neither between the percentage of patients in the intervention group and the supportive care group who achieved the primary or secondary endpoint nor in the median time needed to achieve the first negative PCR sample (p > 0.05).

Conclusion

Prescribing antimalarial medications was not shown to shorten the disease course nor to accelerate the negative PCR conversion rate.

## Introduction

In December 2019, a novel coronavirus named SARS-CoV-2 emerged in China and spread worldwide to be declared by the WHO as a pandemic on March 12, 2020 [1]. Patients with COVID-19 present with fever, dry cough, and shortness of breath; however, some are asymptomatic. The majority of cases have favorable outcomes; nonetheless, older patients and patients with comorbidities may have worse outcomes [2].

SARS-CoV-2 spread mainly through respiratory droplets and close contact. Nevertheless, studies have shown that asymptomatic carriers can be contagious as well [3,4]. The healthcare systems in many countries fall under tremendous pressure of increasing numbers of confirmed cases, and many healthcare authorities recommended two negative nasopharyngeal polymerase chain reaction (PCR) results 24 hours apart before discontinuation of hospital isolation [5,6]. Until mid-2020, there were no therapeutic options approved by the US Food and Drug Administration (FDA) for the prevention or treatment of COVID-19. The current management practices consist of infection prevention and supportive care, such as oxygen supplementation and mechanical ventilation, if needed [7]. Many studies have been conducted to identify effective treatment in order to cure symptomatic patients and limit the transmission to the community. Different medications were proposed to be candidates for the treatment of COVID-19; some of these options focused on the use of old antiviral medications and testing their effectiveness against COVID-19 [8,9]. There are contradictory findings against the effectiveness of antimalarial agents such as hydroxychloroquine (HCQ) and chloroquine on COVID-19. Many studies have demonstrated their effectiveness in inhibiting SARS-CoV-2 [10-12]. In a recent clinical trial, HCQ was reported to cause a significant reduction in viral carriage at day six post inclusion, with around 70% of patients having negative nasopharyngeal PCR sample, compared to untreated patients (12.5%) [8]. On the other hand, a Chinese study reported no significant differences between patients who received HCQ and the control group regarding pharyngeal carriage of viral RNA at day seven [13]. Despite that, there are more than 80 trials registered to investigate the effectiveness of these antimalarial agents against COVID-19 as a monotherapy or in combination with other medications. These trials have poor methodological aspects and reporting [14]. In addition, the use of HCQ might expose some patients to different life-threatening consequences such as cutaneous adverse reactions, fulminant hepatic failure, and ventricular arrhythmias (especially when prescribed with azithromycin) [15-18].

A recent Chinese study focused on the duration of viral shedding and reported a median duration between 20 days and up to 37 days among survivors [19]. Another single-center French study explored the use of the combination of oral HCQ sulfate and azithromycin and reported that this combination was able to negatively convert the nasopharyngeal viral load as tested by PCR in all studied cases on day 12 [20]. Accelerating the negative virus conversion allows for earlier discharge from the hospital and/or designated isolation facilities and facilitates more efficient utilization of the healthcare bed capacity. Previous studies in the Middle East region in general and in Saudi Arabia explored different social, psychological, and clinical outcomes and consequences associated with COVID-19, such as hospitalization related to COVID-19, depression and anxiety, and the social impact of the pandemic on the general population [21-27]. However, there are limited studies that explored the effectiveness of different treatment options including macrolides and antivirals on patients with COVID-19. In this study, we aimed to study the association between negative nasopharyngeal SARS-CoV-2 PCR conversion and different therapeutic interventions (HCQ monotherapy, in combination with macrolide, or in combination with antiviral with or without azithromycin).

This article was previously posted to the medRxiv preprint server on May 11, 2020 (https://doi.org/10.1101/2020.05.08.20095679).

## Materials and methods

Objectives

The objective of this study was to investigate the association between negative nasopharyngeal SARS-CoV-2 PCR conversion and different therapeutic interventions (HCQ monotherapy, in combination with macrolide, or in combination with antiviral with or without azithromycin).

Methods

Setting

This study was conducted from March 7 to April 15, 2020, at the inpatient medical ward of Al Noor Specialist Hospital, a tertiary public hospital in Mecca, Kingdom of Saudi Arabia (KSA).

Study Design

This study is a retrospective cohort study that included 145 patients who were symptomatic and have a PCR confirmed diagnosis of novel coronavirus disease (COVID-19). The Al Noor Specialist Hospital is a designated referral center for confirmed COVID-19 cases in Mecca, KSA. The majority of COVID-19 cases were screened in other facilities and referred to the hospital after being confirmed positive for SARS-CoV-2. All PCR samples are being sent and processed in a regional laboratory. We choose the negative conversion rate of SARS-CoV-2 nasopharyngeal PCR at day five and negative conversion at day 12 from the first positive sample as our primary and secondary endpoints as it was shown to adequately measure treatment effectiveness in the studies of Gautret et al. [8,20]. During the time of conducting this study, the Saudi Ministry of Health (MOH) recommended interventions for confirmed cases only; thus, all patients were receiving the best supportive care only until positive PCR results are confirmed. Furthermore, the MOH guideline suggests (optional) starting antiviral and/or HCQ after the approval of an infectious disease consultant.

Study Population

We included all PCR confirmed cases of SARS-CoV-2 admitted to the medical ward of Al Noor Specialist Hospital. The hospital protocol of retesting of symptomatic patients follows the Saudi Center for Disease Prevention and Control recommendations, which recommends retesting when a patient is clinically recovered and to be repeated every 72 hours if the result remains positive [6]. Our exclusion criteria were as follows: 1) patients less than 12 years of age, 2) cases that were directly admitted to the intensive care unit (ICU), 3) patients who develop critically severe disease and were shifted to the ICU while still showing a positive nasopharyngeal PCR results for SARS-CoV-2, and 4) clinically stable patients who were transferred to the Ministry of Health (MOH)-designated isolation facilities while still having positive PCR results. The second and third exclusion criteria were defined because the retesting protocol at the ICU is performed irregularly and with long interval periods.

Outcomes

The primary endpoint of the study is achieving negative SARS-CoV-2 nasopharyngeal PCR within five days or less from the start of the intervention. The secondary endpoint was achieving a negative sample within 12 days or less from the first positive PCR result.

Intervention and Control Groups

The patients were categorized into two main groups. Group A included patients who received any active interventions. We defined patients in active interventions as those who received any of the following medications: chloroquine, HCQ, ribavirin, and/or lopinavir and ritonavir. On the other hand, group B is defined as the best supportive care group and included patients who did not receive any dose of the active interventional drugs.

**Figure 1:**
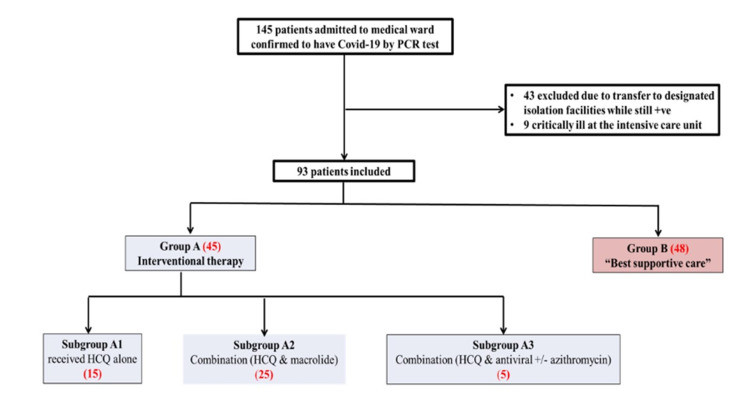
Study flowchart

Group A was subsequently subgrouped into A1, A2, and A3. Subgroup A1 included patients who received any dose of chloroquine (600 mg at diagnosis, followed by 300 mg 12 hours later BID for five to seven days) or HCQ (400 mg every 12 hours in the first day, followed by 200 mg twice daily for five to seven days) without any dose of azithromycin antibiotics or antivirals (ribavirin and/or lopinavir and ritonavir). Subgroup A2 included patients who received any dose of chloroquine or HCQ and any dose of azithromycin (500 mg once daily) not necessary simultaneously. Subgroup A3 (multiple intervention subgroups) includes patients who received any dose of chloroquine or HCQ and any dose of any antiviral drugs (ribavirin 400 mg every 12 hours, and/or lopinavir and ritonavir 400/100 mg every 12 hours) with or without azithromycin. Figure 1 presents the study flowchart.

Definitions

The severity of the disease was defined as follows: 1) patients with mild disease were defined as those with upper respiratory tract symptoms (rhinorrhea, sore throat, headache, myalgia, body pain, low-grade fever, and/or dry cough) with the absence of a clinical or radiological finding of pneumonia; 2) patients with moderate disease were defined as symptomatic patients with either clinical or radiological sign of pneumonia; 3) patients with severe disease were defined as those with confirmed COVID-19 pneumonia, with any of the following: respiratory rate ≥ 30/minute, blood oxygen saturation ≤ 93% at rest, PaO_2_/FiO_2_ ratio < 300, and lung infiltration > 50% of the lung field; and 4) patients with critically severe disease were defined with any of the following: respiratory failure requiring invasive mechanical ventilation, shock, or organ failure requiring admission to the intensive care unit.

Negative conversion: We used the first negative nasopharyngeal PCR test to define the negative conversion.

Time to negativity definition: Days to negative PCR clearance were calculated from the first positive sample to the first negative sample. Days between starting the medical management to the achievement of PCR negative clearance were calculated from the first dose of HCQ or chloroquine in subgroups A1 and A2. In subgroup A3, it was calculated from the first dose of any given intervention, whether the antimalarial drug or the antiviral medications, whichever first. Regarding group B, it was calculated starting from the date of admission to the date of negative PCR clearance. As a referral center, the admission date lagged behind the first positive PCR results.

Ethical Approval

The study protocol was reviewed and approved by the institutional ethics board of the Ministry of Health of Saudi Arabia (H-02-K-076-0420-286) on February 11, 2020. All the recruited subjects provided written consent. 

Statistical Analysis

Data were analyzed using the SPSS software version 25 (IBM Corp., Armonk, NY, USA). The descriptive analysis was reported as mean (±standard deviation (SD)) for normally distributed quantitative variables and as median (interquartile range (IQR)) for non-normally distributed quantitative variables. Kolmogorov-Simonov, Shapiro-Wilk, and histogram tests were used to check the normality of the data. Categorical data were reported as percentages and frequencies. The Mann-Whitney U test/Kruskal-Wallis test was used to compare the median days to achieve PCR clearance between different demographic groups. In addition, logistic regression analysis was applied to identify factors associated with PCR clearance. A confidence interval (CI) of 95% (p < 0.05) was applied to represent the statistical significance of the results, and the level of significance was assigned as 5%.

## Results

Baseline characteristics

A total of 93 out of 145 patients (64.1%) who have met the inclusion criteria were included in this study. Of the 52 patients who were excluded, 43 were clinically stable and were transferred to the MOH-designated isolation facilities while still having positive PCR results. Nine patients were excluded as they were critically ill and shifted to the ICU.

A total of 45 patients (48.4%) formed the intervention group (group A), while 48 patients met the criteria of the best supportive care group. From the intervention group A, 15, 25, and five patients met the criteria for subgroups A1, A2, and A3, respectively. The majority of the patients in the intervention group were males (n = 27, 60%); contrarily, the majority of the patients in group B were females (n = 26, 54.2%). Group A had significantly more severe disease, with nine patients (20%) presenting with severe illness compared with one patient (2.1%) in group B (p < 0.000). The majority of the patients who received best supportive care had mild disease (n = 41, 85.4%). Of the patients with moderately severe illness, 44.4% and 12.5% were in groups A and B, respectively. Table 1 presents patient baseline demographics.

**Table 1:** Patient baseline characteristics SD: standard deviation

Variable	Frequency (%)
Age (years), mean (SD)	43.9 (15.9)
Group A age (years), mean (SD)	
Subgroup A1	42.9 (17.2)
Subgroup A2	46.4 (16.4)
Subgroup A3	41.4 (18.3)
Group B age (years), mean (SD)	43.2 (15.4)
Gender	
Overall (male), number (%)	49 (52.7)
Group A	27 (60)
Group B	22 (45.8)
Severity for all patients, number (%)	
Mild	57 (61.3)
Moderate	26 (28)
Severe	10 (10.8)
Severity for group A, number (%)	
Mild	16 (35.6)
Moderate	20 (44.4)
Severe	9 (20)
Severity for group B, number (%)	
Mild	41 (85.4)
Moderate	6 (12.5)
Severe	1 (2.1)

Effect of interventions

As shown in Figure 1, subgroup A1 patients received HCQ, and subgroup A2 patients received a combination of HCQ and macrolide. All patients (n = 5) in subgroup A3 received azithromycin and HCQ. Three patients received lopinavir/ritonavir combined with ribavirin, while two have received it without ribavirin.

All patients in this study needed a median time of six days (IQR: 4.50-9.00) from the first positive to the first negative PCR sample and a median time of three days (IQR: 2.00-6.50) from the time of starting the intervention to the first negative PCR sample. Around 71% (n = 66) and 81.7% (n = 76) of patients achieved the primary and secondary endpoints, respectively. There was no statistically significant difference between the percentage of patients in the intervention group (group A) and the supportive care group (group B) who achieved the primary or secondary endpoint (p > 0.05). In group A, 73.3% (n = 33) achieved the primary endpoint, and 84.4% (n = 38) achieved the secondary endpoint. A smaller percentage of patients (68.8% (n = 33) and 79.2% (n = 38)) achieved the primary and secondary endpoints in group B. There was no statistically significant difference in the median time to negative conversion from the first positive to the first negative PCR sample or from the time of starting the intervention between the two groups (p > 0.05) (Table 2).

**Table 2:** Median time and percentage of patients who achieved the primary and secondary outcomes

Variable	All patients	Group A	Group B	P-value
Median time from the first positive to the first negative PCR sample for all patients (IQR)	6.00 (4.50–9.00)	6.00 (5.00–9.00)	6.00 (4.00–9.75)	0.574
Median time from intervention to first negative PCR sample for all patients (IQR)	3.00 (2.00–6.50)	3.00 (2.00–6.50)	3.00 (2.00–6.75)	0.895
Patients achieved primary endpoint and had negative PCR sample within five days or less (number (%))	66 (71)	33 (73.3)	33 (68.8)	0.655
Patients achieved secondary endpoint and had negative PCR sample within 12 days or less (number (%))	76 (81.7)	38 (84.4)	38 (79.2)	0.597

Patient characteristics and PCR clearance time

Table 3 details the median time needed to achieve the first negative PCR sample from the first positive and from intervention stratified by patients’ demographics and treatment groups. There was a statistically significant difference in the median time from the first positive and intervention to the first negative PCR sample between elderly patients (aged 45 years and above) and younger patients (p < 0.01). In addition, the median time from intervention to the first negative PCR sample significantly differed by disease severity (p < 0.05). On the other hand, there was no statistically significant difference between the intervention group (group A) and the supportive care group (group B) in terms of the median time from the first positive and intervention to the first negative PCR sample (p > 0.05).

**Table 3:** Patient characteristics and PCR clearance time *p < 0.05, ** p < 0.01 IQR: interquartile range

Variable	Median time from intervention to first negative sample (IQR)	P-value	Median time from first positive to first negative sample (IQR)	P-value
Age				
Below 45 years	3.00 (1.00–4.00)	0.005**	6.00 (4.00–7.00)	0.003**
45 years and above	4.50 (2.00–11.25)		8.00 (5.00–14.00)	
Gender				
Male	3.00 (2.00–5.00)	0.172	6.00 (4.00–8.00)	0.095
Female	3.50 (1.00–9.00)		7.00 (5.00–13.00)	
Severity				
Mild	3.00 (1.00–5.50)	0.039*	6.00 (4.00–9.00)	0.103
Moderate	4.00 (2.00–6.25)		6.50 (5.00–9.00)	
Severe	6.50 (3.75–9.00)		10.50 (5.00–15.25)	
Treatment group				
Group A	3.00 (2.00–6.50)	0.473	6.00 (5.00–9.00)	0.632
Subgroup A1	3.00 (2.00–6.00)		6.00 (4.00–9.00)	
Subgroup A2	3.00 (2.00–6.00)		6.00 (5.00–8.50)	
Subgroup A3	7.00 (4.00–9.50)		13.00 (5.00–14.50)	
Group B	3.00 (2.00–6.75)		6.00 (4.00–9.75)	

Binary logistic regression analysis showed that males were 3.9 times more likely to achieve the primary endpoint and achieve a negative PCR sample within five days compared with females (OR: 3.90, 95%CI: 1.49-10.22). In addition, males were 4.7 times more likely to achieve the secondary endpoint and achieve a negative PCR sample within 12 days compared with females (OR: 4.71, 95%CI: 1.41-15.83). On the other hand, age was negatively associated with achieving the primary and secondary endpoints, and elderly patients aged 45 years and above were less likely to achieve them by around 77% (OR: 0.23, 95%CI: 0.09-0.59) and 93% (OR: 0.07, 95%CI: 0.02-0.35), respectively.

Using multiple logistic regression, we applied two models: the first one to explore the effect of the intervention subgroups (A1, A2, and A3) compared with supportive care group (group B) on achieving the primary endpoint (achieving negative PCR sample within five days or less) adjusting for age, gender, and disease severity and the second model to explore the effect on achieving the secondary endpoint (achieving negative PCR sample within 12 days or less). The first model did not find any statistically significant difference between any intervention subgroup and the supportive care group in achieving the primary endpoint (p > 0.05). The second model found a negative association between the intervention subgroup A3 and achieving the secondary endpoint. Patients in subgroup A3 were around 97% less likely to achieve the secondary endpoint (OR: 0.033, 95%CI: 0.001-0.863). However, the association was very weak (p = 0.040).

## Discussion

This was a retrospective cohort study of patients who were admitted with a PCR confirmed diagnosis of COVID-19. We investigated the association between negative nasopharyngeal SARS-CoV-2 PCR clearance and different therapeutic interventions. This study found that the use of HCQ for the treatment of COVID-19 whether as monotherapy or in combination therapy was not significantly associated with better negative PCR clearance or shorter time compared with supportive care. Males were four to five times more likely to achieve negative PCR clearance compared with females within five days and 12 days, respectively. In addition, age was negatively associated with achieving negative PCR clearance within five days and 12 days.

To date, there is no proven effective therapy for SARS-CoV-2; however, HCQ was adopted as an optional therapy after an encouraging initial in vitro result [12]. Despite the lack of convincing evidence of its efficacy, HCQ has been suggested to be used by different medical regularity authorities based on small, non-randomized promising clinical studies [20-28].

The findings of our study align with previous studies that demonstrated no superior value for the administration of HCQ in treating COVID-19 [13,14]. The baseline demographic characteristics in our study showed comparable age across the two groups. However, group A (treated using HCQ, with or without azithromycin and additional antiviral therapy) was more likely to be males (60%). Moreover, group A, especially subgroup A3, had more severe disease. There was no statistically significant difference in the median time required to the first negative PCR sample between group A and group B (p > 0.05). Compared to the study of Gautret et al., where the number of contagious patients dropped to zero on day 12 [8], 18.3% of our patients who received interventions remained positive after the 12th day. Despite that there was a difference in the baseline severity of the cases between patients who received pharmacological therapy (using HCQ as monotherapy or combination therapy) (group A) and patients who received supportive care (group B), we did not find any statistically significant difference in the rate of achieving negative PCR clearance or in the time needed to achieve it (p > 0.05). There was no statistically significant difference between the percentage of patients in group A who achieved primary and secondary endpoint (73.3% and 84.4%) compared with group B (68.8% and 79.2%) (p > 0.05).

Our study showed that the intervention group did not have a shorter disease course nor faster negative conversion of the nasopharyngeal swab result for SARS-CoV-2. We did not study the clinical usefulness of these interventions in terms of clinical improvements, such as attenuation of the disease severity or mortality reduction. However, we are aware of the negative study that showed no reduction in mechanical ventilation risk in patients hospitalized with COVID-19 and who received HCQ, either with or without azithromycin [29].

Despite that, the FDA has approved and authorized a number of COVID-19 therapies for mild, moderate, and severe diseases. COVID-19 prevention is more successful than COVID-19 treatment. Vaccines help people avoid becoming sick or becoming very ill. Evidence suggests that immunizations are quite successful at avoiding COVID-19-related severe illness, hospitalization, and mortality, including the Alpha and Delta variants. People who have been fully vaccinated are less likely to become seriously ill. According to studies, vaccination produces a stronger immune response than the one produced by COVID-19 infection. Vaccination after an infection has significantly more advantages than any known or possible risk [30].

This study has several strengths. To our knowledge, this was the first study in the Middle East region to explore the association between the use of HCQ as monotherapy or combination therapy and the odds of achieving negative PCR samples in patients with COVID-19. As the data collection center was the designated regional COVID-19 center, patients were diagnosed at different locations and then transferred to our center; thus, our study cohort is less susceptible to selection biases of single-center studies. However, our study has some limitations. The study design was a retrospective single referral center. Thus, it inherent all retrospective analysis limitations, such as non-randomization of treatments. In addition, the small sample size in our study, specifically for the subgroups in the intervention group (group A), might have limited our ability to explore statistically significant differences and had led to wide confidence intervals. Although we conducted multiple logistic regression to adjust for the severity of the disease and the age, patients could still have confounders that we were not able to measure and might influence our findings.

## Conclusions

This study showed no significant difference in time to negative PCR clearance between patients who received HCQ whether alone or in combination with azithromycin and/or antivirus drugs compared with patients treated with best supportive care. Prescribing antimalarial medications was not shown to shorten the disease course nor to accelerate the negative PCR conversion rate or hospital discharge.
